# Detraining after short‐term exercise induces hyperphagia and obesity with fatty liver and brown adipose tissue whitening in young male OLETF rats

**DOI:** 10.14814/phy2.16055

**Published:** 2024-06-14

**Authors:** Takaya Oshima, Kaho Takaishi, Misuzu Nishihira, Son Tien Nguyen, Susumu Urakawa, Haruya Ohno, Naoto Fujita

**Affiliations:** ^1^ Department of Musculoskeletal Functional Research and Regeneration, Graduate School of Biomedical and Health Sciences Hiroshima University Hiroshima Japan; ^2^ Department of Molecular and Internal Medicine, Graduate School of Biomedical and Health Sciences Hiroshima University Hiroshima Japan; ^3^ Department of Bio‐Environmental Adaptation Sciences, Graduate School of Biomedical and Health Sciences Hiroshima University Hiroshima Japan

**Keywords:** detraining, exercise, fat accumulation, hyperphagia, obesity at a young age

## Abstract

This study examined the effects of exercise and detraining at a young age on fat accumulation in various organs. Four‐week‐old male Otsuka Long‐Evans Tokushima Fatty (OLETF) rats were assigned to either the non‐exercise sedentary (OLETF Sed) or exercise groups. The exercise group was subdivided into two groups: exercise between 4 and 12 weeks of age (OLETF Ex) and exercise between 4 and 6 weeks of age followed by non‐exercise between 6 and 12 weeks of age (OLETF DT). Body weight was significantly lower in the OLETF Ex group than in the OLETF Sed group at 12 weeks of age. Fat accumulation in the epididymal white adipose tissue, liver, and brown adipose tissue was suppressed in the OLETF Ex group. During the exercise period, body weight and food intake in the OLETF DT group were significantly lower than those in the OLETF Sed group. However, food intake was significantly higher in the OLETF DT group than in the OLETF Sed group after exercise cessation, resulting in extreme obesity with fatty liver and brown adipose tissue whitening. Detraining after early‐onset exercise promotes hyperphagia, causing extreme obesity. Overeating should be avoided during detraining periods in cases of exercise cessation at a young age.

## 1 INTRODUCTION

Childhood obesity carrying over to adulthood has emerged as an important issue in recent years. According to a study conducted in 2017, childhood obesity rates worldwide have risen 10‐fold in the last 40 years, and the number of cases has reached 124 million (NCD‐RisC. Worldwide trends in body‐mass index, underweight, overweight, and obesity from, 1975). Overweight and obesity in children and adolescents are linked to a high probability of adulthood obesity (Padez et al., 2005). Ahmad et al. (2010) reported that 80% of obese adolescents aged 10–14 years, 50% of obese children aged 6–9 years, and 25% of obese children aged <5 years remain obese in adulthood. Childhood obesity increases the risk of early‐onset of obesity‐related health problems such as type 2 diabetes and early mortality in adulthood (Lloyd et al., 2012; The et al., 2010). Therefore, to reduce obesity‐related health problems in adulthood, it is desirable not to carry over childhood obesity to adulthood.

Regular physical exercise by obese children and adolescents promotes a decrease in adiposity and prevents adulthood obesity (Kelley & Kelley, 2013; Paes et al., 2015). In addition, aerobic and resistance exercises lead to a significant reduction in ectopic fat accumulation in organs such as the liver in obese children (Lee et al., 2012, 2013; van der Heijden et al., 2010). Several studies have recommended increasing the amount of physical activity earlier in life, such as in the developmental period, to suppress obesity (Aggoun, 2007; Patterson et al., 2008; Schroeder et al., 2010). However, increased sedentary behavior in children and adolescents has become a global issue that leads to childhood obesity. According to a British study (Janssen et al., 2016), the amount of sedentary time spent by children and adolescents aged >7 years has increased substantially. During the waking day, over 50% of the time was sedentary for 7‐year‐olds. Moreover, sedentary time increased in 9‐, 12‐, and 15‐year‐olds and exceeded 75% of waking days by the 15‐year mark (Janssen et al., 2016). In addition, a systematic review by Tanaka et al. reported that the accelerometer‐measured daily sedentary time increased around the age of school entry and then rose incrementally with age (Tanaka et al., 2014). A recent epidemiological survey showed insufficient physical activity in 81% of students aged 11–17 years (Guthold et al., 2020). Thus, decreased physical activity and increased sedentary behavior beginning at school age are predominant factors causing childhood obesity. Although it is known that weight loss resulting from increased physical activity is diminished by exercise cessation during detraining (Mitsuhashi et al., 2011), only a few studies have investigated the influence of childhood exercise cessation on metabolic characteristics later in life.

Low levels of physical activity cause energy intake to consistently exceed energy expenditure, leading to triacylglycerolemia and subsequent fat accumulation in multiple organs (Olsen et al., 2008). Excess fat accumulates not only in the white adipose tissue but also in the liver, pancreas, and skeletal muscle, thus causing dysfunction in several organs (Morelli et al., 2013). Moreover, ectopic fat accumulation is associated with an increased risk of developing metabolic and inflammatory diseases (Jacobs et al., 2016). Chronic ectopic fat accumulation induces nonalcoholic fatty liver disease, fatty pancreas, and increased intramuscular fat deposition. These in turn contribute to glucose intolerance, impaired insulin secretion, and insulin resistance (Saponaro et al., 2015). Excess fat also accumulates in brown adipose tissue (BAT); this abnormal fat collects as unilocular white adipocytes instead of the normal multilocular brown adipocyte pattern. BAT whitening with unilocular transformation decreases energy consumption through impaired uncoupling of oxidative phosphorylation and accelerates the progression of obesity (Nishimoto & Tamori, 2017; Thompson et al., 2013). Regular physical exercise is expected to reduce visceral fat. However, the effectiveness of exercise on ectopic fat accumulation in childhood obesity remains unclear. Exercise always leads to weight loss in adult obesity. However, hard exercise with resulting body weight loss is not encouraged in childhood obesity from the perspective of promoting physical development. Therefore, the influence of regular exercise and detraining on fat accumulation may differ between childhood and adulthood. An enhanced understanding of the scientific influence of exercise and detraining at a young age on visceral fat could contribute to the suppression and prevention of childhood obesity transitioning to adulthood obesity. The aim of the present study was to identify therapeutic targets for childhood obesity by examining the influence of exercise and detraining on fat accumulation in various organs at an early age.

## 2 MATERIALS AND METHODS

### 2.1 Animals and diet

Four‐week‐old male Otsuka Long‐Evans Tokushima Fatty (OLETF) rats and age‐matched male Long‐Evans Tokushima Otsuka (LETO) rats were used as spontaneously obese and non‐obese animals, respectively.

All rats were fed a standard diet composed of 7.9% moisture, 23.1% crude protein, 5.1% crude fat, 5.8% crude ash, 2.8% crude fiber, and 55.3% nitrogen‐free extract (Oriental Yeast, Tokyo, Japan). Food and water were provided ad libitum. Three rats were placed in a cage and housed in a controlled room with a 12‐h light–dark cycle (light on from 8:00 and off from 20:00) at a constant temperature of 22 ± 2°C. This study was approved by the Institutional Animal Care and Use Committee of Hiroshima University (A19‐163) and conducted in accordance with the Hiroshima University Regulations for Animal Experimentation. All experiments were conducted in accordance with the National Institute of Health Guidelines for the Care and Use of Laboratory Animals.

*Experiment 1: Influence of exercise and detraining at a young age on fat accumulation in various organs*.

**FIGURE 1:**
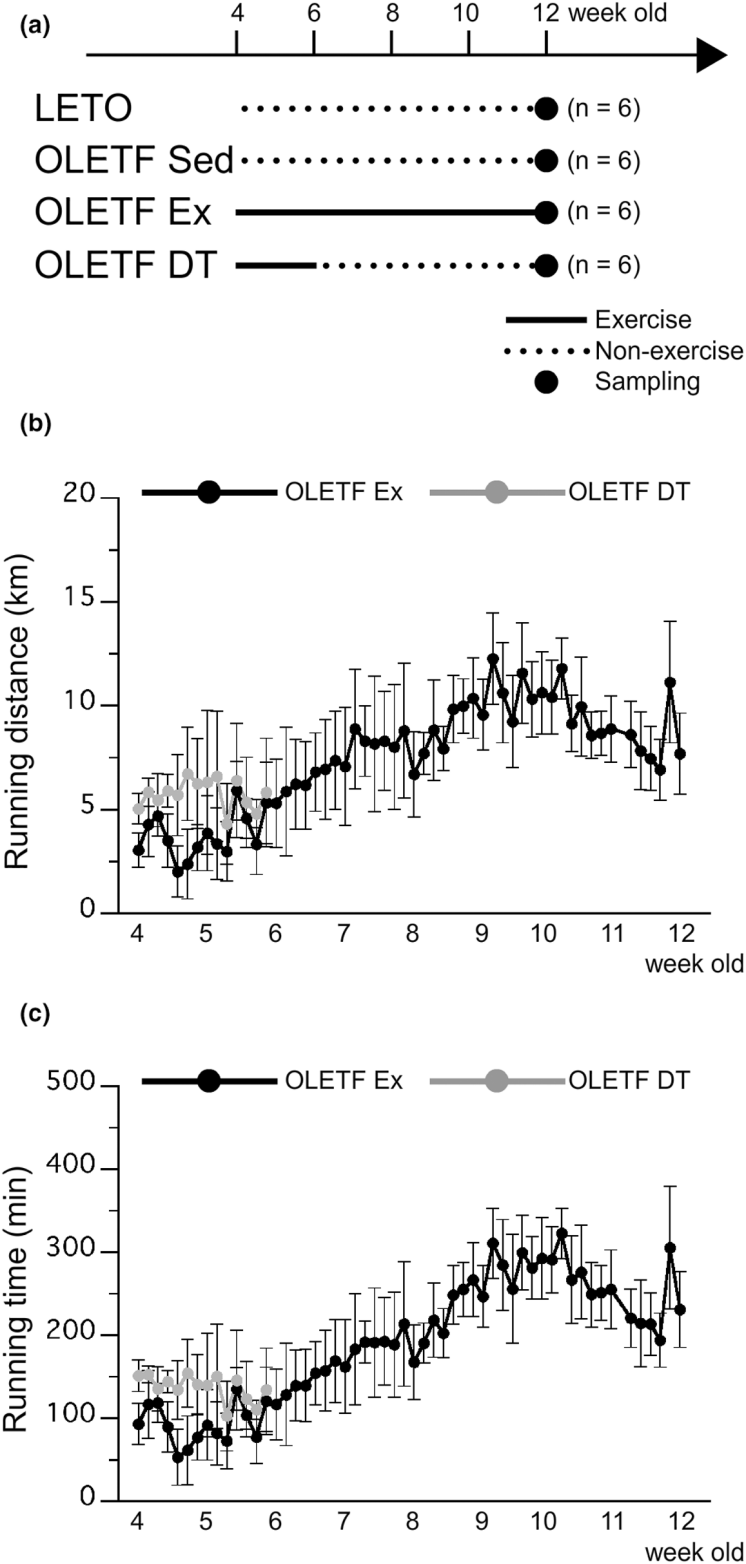
Experimental design and exercise in the OLETF Ex and OLETF DT groups. (a) Experimental design. LETO, Long‐Evans Tokushima Otsuka rat; OLETF, Otsuka Long‐Evans Tokushima Fatty rat; OLETF Sed, OLETF non‐exercise sedentary; OLETF Ex, OLETF exercise from 4 to 12 weeks of age; OLETF DT, OLETF exercise from 4 to 6 weeks of age and detraining from 6 to 12 weeks of age. Solid and dashed lines indicate exercise and non‐exercise periods, respectively. Dark circles indicate the point of tissue sampling. (b) Daily total running distance. (c) Daily total running time. Values represent means ± standard deviation.

The OLETF rats were randomly assigned to either the non‐exercise sedentary (OLETF Sed, *n* = 6) or to the exercise (*n* = 12) group. Based on the exercise period, the OLETF rats in the exercise group were subdivided into two groups: exercise between 4 and 12 weeks of age (OLETF Ex, *n* = 6) and exercise between 4 and 6 weeks of age followed by non‐exercise detraining between 6 and 12 weeks of age (OLETF DT, *n* = 6). LETO rats as non‐obese animals were assigned to the only non‐exercise sedentary group (LETO, *n* = 6, Figure 1a).

*Experiment 2: Influence of food restriction during the detraining period*.

The OLETF rats were randomly assigned to either the non‐exercise sedentary (OLETF Sed, *n* = 6) or to the detraining (*n* = 12) group [exercise between 4 and 6 weeks of age followed by non‐exercise detraining between 6 and 12 weeks of age]. The detraining group was further subdivided into non‐food restriction (OLETF DT, *n* = 6) and food restriction (OLETF DTFR, *n* = 6) groups. The rats in the OLETF DT group were fed ad libitum during the detraining period, whereas food intake in the OLETF DTFR group during this period was similar to that in the OLETF Sed group. Measurement of food intake was performed twice a day 8:00 and 20:00. At the same time, the cage in the OLETF DTFR group was provided the same amount of food as age‐matched OLETF Sed group.

### 2.2 Exercise training protocol

OLETF rats in the exercise groups were individually housed in cages with a freely accessible running wheel for 12 h (20:00–8:00). A magnetic sensor attached to the cage recorded the wheel running time and distance for 12 h. Food and water were provided ad libitum in cages with running wheels.

### 2.3 Tissue sampling

The rats in each group were euthanized with an overdose of sodium pentobarbital at 12 weeks of age. Blood samples were collected 5 h after exercise from the caudal vena cava, and tissue samples of the epididymal white adipose tissue (eWAT), liver, pancreas, BAT from both the interscapular regions, and gastrocnemius muscle were immediately obtained at each week of age. The liver and gastrocnemius muscles were weighed. They were then frozen in liquid nitrogen and stored at −80°C until further analysis. The eWAT, BAT and pancreatic samples were weighed and fixed with 4% paraformaldehyde in 0.1 M phosphate buffer.

### 2.4 Blood analysis

The blood samples were centrifuged at 3000 rpm for 10 min at room temperature (22 ± 2°C). Plasma was collected to determine the free fatty acid (FFA) and triacylglycerol (TAG) concentrations. FFA and TAG levels were measured using commercially available spectrophotometric assay kits (294‐63601 and 290‐63701, respectively; Wako, Osaka, Japan) according to the manufacturer's instructions.

### 2.5 Histological analysis

The eWAT and BAT embedded in paraffin were sliced into a thickness of 5 μm sections and stained with hematoxylin and eosin (HE) for histological observation. The diameters of 200 white adipocytes per rat in three or four randomly chosen fields were measured using the ImageJ software (NIH, MD, USA).

The left lateral lobe of the liver was sliced to a section thickness of 10 μm using a cryostat and stained with oil red O to visualize the localization of ectopic fat accumulation. Additionally, total lipid levels from the liver samples were extracted using the Folch method. The TAG concentrations were measured using commercially available spectrophotometric assay kits (290‐63701, Wako), adhering to the manufacturer's instructions.

The pancreatic head was embedded in paraffin, sliced to a section thickness of 5 μm, and stained with HE for histological observation. Images were used to observe the localization of ectopic fat accumulation in the exocrine and endocrine portions, including the pancreatic islets.

Transverse sections from the middle part of the lateral head of the gastrocnemius muscle at a thickness of 10 μm were prepared using a cryostat. Although the portion was characterized as mixed red and white muscle type macroscopically, white muscle types were the majority. The sections were stained with oil red O to observe the localization of intramuscular lipids. Briefly, the frozen sections were fixed with 4% formaldehyde for 1 h at room temperature. The sections were rinsed in deionized water and incubated in a solution of oil red O (40491, Muto Pure Chemicals, Tokyo Japan) for 1 h at room temperature. After rinsing with deionized water, the sections were embedded in glycerol gelatin. Serial transverse sections were also stained for adenosine triphosphatase (ATPase, pH 4.1) and succinate dehydrogenase (SDH) activities to categorize the muscle fibers as type I, IIA, or IIB (Matsumoto et al., 2014). For ATPase staining, the sections were preincubated in barbital‐acetate buffer (pH 4.1) for 5 min at room temperature and incubated in 0.1 M barbital buffer containing 0.18 M CaCl_2_ and 4 mM ATP (pH 9.4) for 45 min at room temperature. Following the incubation, the sections were washed with 1% CaCl_2_ and 2% CoCl_2_ every 3 min. After washing with 0.01 M sodium barbital, the sections were visualized using 1% ammonium sulfide. For SDH staining, the sections were incubated in 0.1 M phosphate buffer (pH 7.4) containing 0.1% nitro blue tetrazolium and 0.1 M sodium succinate for 45 min at 37°C. Muscle fibers densely stained for both ATPase (pH 4.1) and SDH were categorized as type I; those stained lightly for ATPase (pH 4.1) and densely for SDH were categorized as type IIA; and those stained lightly for both ATPase (pH 4.1) and SDH were categorized as type IIB.

### 2.6 Statistical analysis

Data are expressed as the mean ± standard deviation. Differences between groups were evaluated using either two‐way or one‐way analysis of variance with Bonferroni's post hoc test. Statistical significance was set at *p* < 0.05. All statistical analyses were performed using the SPSS statistical analysis software (IBM SPSS Statistics version 19.0, IBM Japan, Tokyo, Japan).

## 3 RESULTS

### 3.1 Changes in body weight and food intake

**FIGURE 2:**
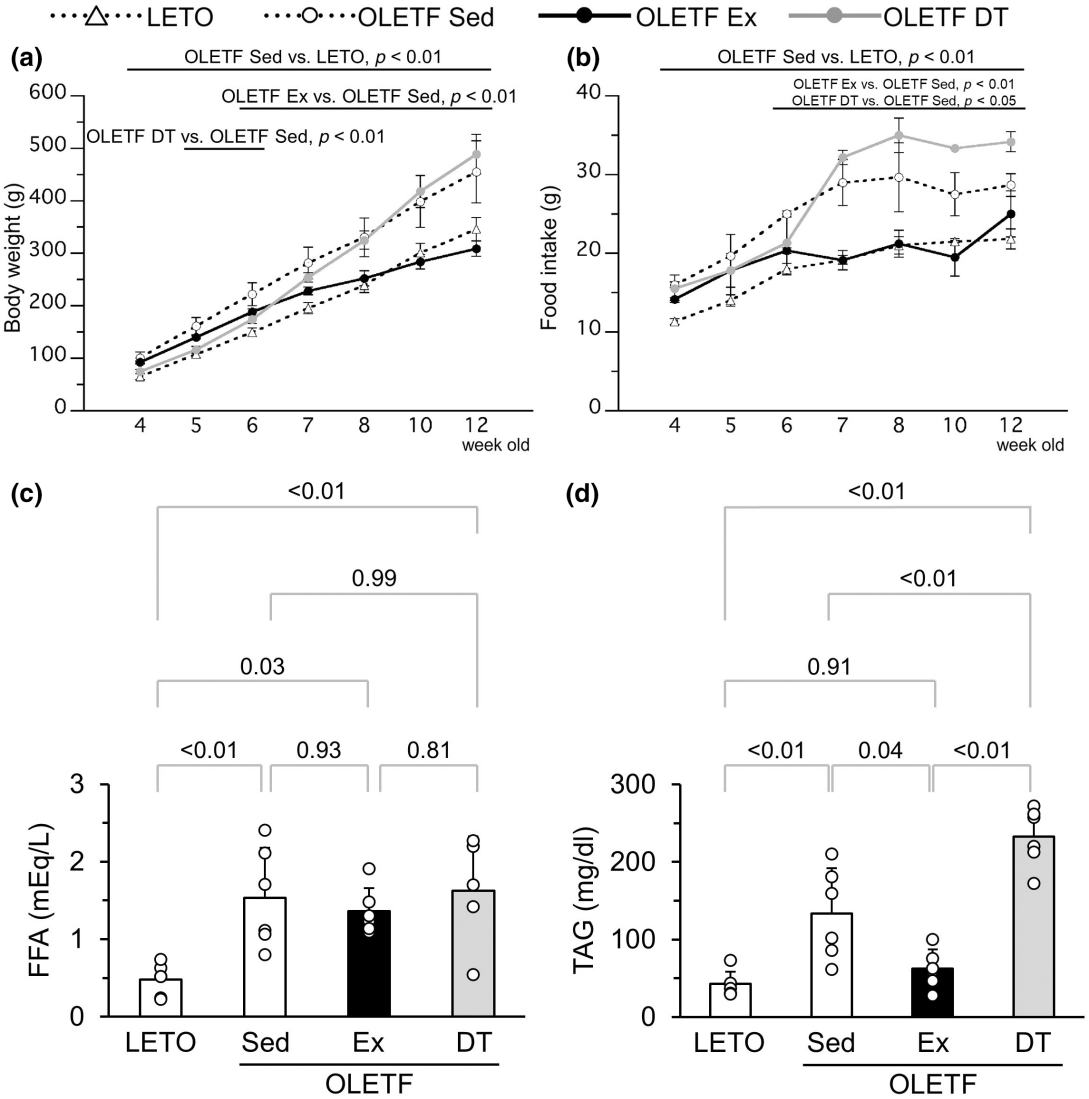
Influences of exercise and detraining at a young age on obesity. (a) Changes in body weight. (b) Daily food intake in a rat. (c) Plasma FFA levels at 12 weeks of age. (d) Plasma TAG levels at 12 weeks of age. FFA, free fatty acid; TAG, Triacylglycerol. Values represent means ± standard deviation. *p*‐Values were shown in the graph.

Body weight in the OLETF Sed group gradually increased throughout the experimental period. The weight was significantly higher in the OLETF Sed group than in LETO rats after 4 weeks of age (Figure 2a). Moreover, food intake was significantly higher in the OLETF Sed group than in LETO rats throughout the experimental period (Figure 2b).

Body weight in the OLETF Ex group slowly increased throughout the experimental period and was significantly lower than that in the OLETF Sed group after 6 weeks of age (Figure 2a). Food intake was significantly lower in the OLETF Ex group than in the OLETF Sed group after 6 weeks of age (Figure 2b).

Body weight in the OLETF DT group was significantly lower than that in the OLETF Sed group between 5 and 6 weeks of age, that is, during the exercise period (Figure 2a). Food intake in the OLETF DT group was significantly lower than that in the OLETF Sed group at 6 weeks of age during the exercise period (Figure 2b). Body weight in the OLETF DT group significantly increased during the detraining period compared with the exercise period, and there were no significant differences in body weight between the OLETF DT and OLETF Sed groups between 7 and 10 weeks of age (i.e., detraining for 1–4 weeks) (Figure 2a). Interestingly, food intake in the OLETF DT group significantly increased during the detraining period compared with the exercise period and was significantly higher than that observed in the OLETF Sed group after 7 weeks of age (Figure 2b). In addition, body weight tended to be higher in the OLETF DT group than in the OLETF Sed group at 12 weeks of age (*p* = 0.059) (Figure 2a).

### 3.2 Running distance and running time

The running distance (Figure 1b) and running time (Figure 1c) gradually increased 2 weeks after the onset of exercise. These values continued to increase throughout the exercise period in the OLETF Ex group.

### 3.3 Plasma FFA and TAG levels

Plasma FFA levels were significantly higher in the OLETF Sed group than in LETO rats (Figure 2c). There were no significant differences in plasma FFA levels between the OLETF Sed, OLETF Ex, and OLETF DT groups.

Plasma TAG levels were significantly higher in the OLETF Sed group than in LETO rats (Figure 2d). Plasma TAG levels in the OLETF Ex group were significantly lower than those in the OLETF Sed group. Conversely, the values in the OLETF DT group were significantly higher than those in the OLETF Sed group.

### 3.4 eWAT

**FIGURE 3:**
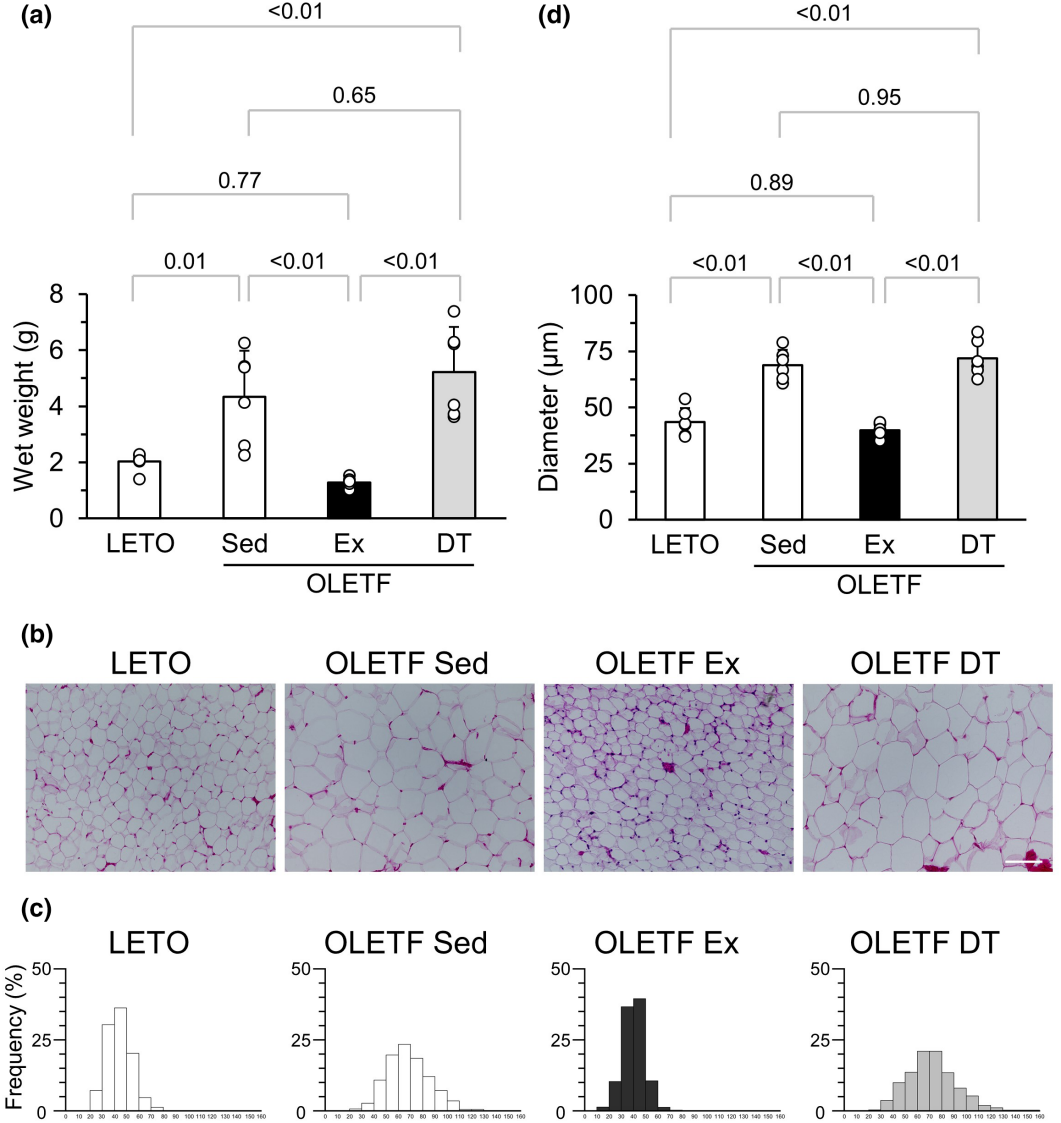
Influences of exercise and detraining at a young age on eWAT. (a) Wet weight of eWAT. (b) Representative sections of eWAT stained with hematoxylin and eosin. (c) Distribution of the white adipocyte diameter. (d) Mean adipocyte diameter. Data are shown at 12 weeks of age. eWAT, epididymal white adipose tissue. Values represent means ± standard deviation. *p*‐Values were shown in the graph. Bar = 100 μm.

The wet weight of eWAT was significantly higher in the OLETF Sed group than in LETO rats (Figure 3a). The wet weight of eWAT in the OLETF Ex group was significantly lower than that in the OLETF Sed group. There was no significant difference in the wet weight of eWAT between the OLETF DT and OLETF Sed groups.

Hypertrophied adipocytes were frequently observed in the OLETF Sed and OLETF DT groups but rarely in LETO rats and the OLETF Ex group (Figure 3b). The distribution peaks of adipocyte diameter in eWAT were 60–70, 40–50, and 60–70 μm in the OLETF Sed, OLETF Ex, and OLETF DT groups, respectively (Figure 3c). The mean diameter of adipocytes was significantly larger in the OLETF Sed group than in LETO rats (Figure 3d). The mean diameter of adipocytes in the OLETF Ex group was significantly smaller than that in the OLETF Sed group. There were no significant differences in the mean adipocyte diameter between the OLETF DT and OLETF Sed groups.

### 3.5 BAT

**FIGURE 4:**
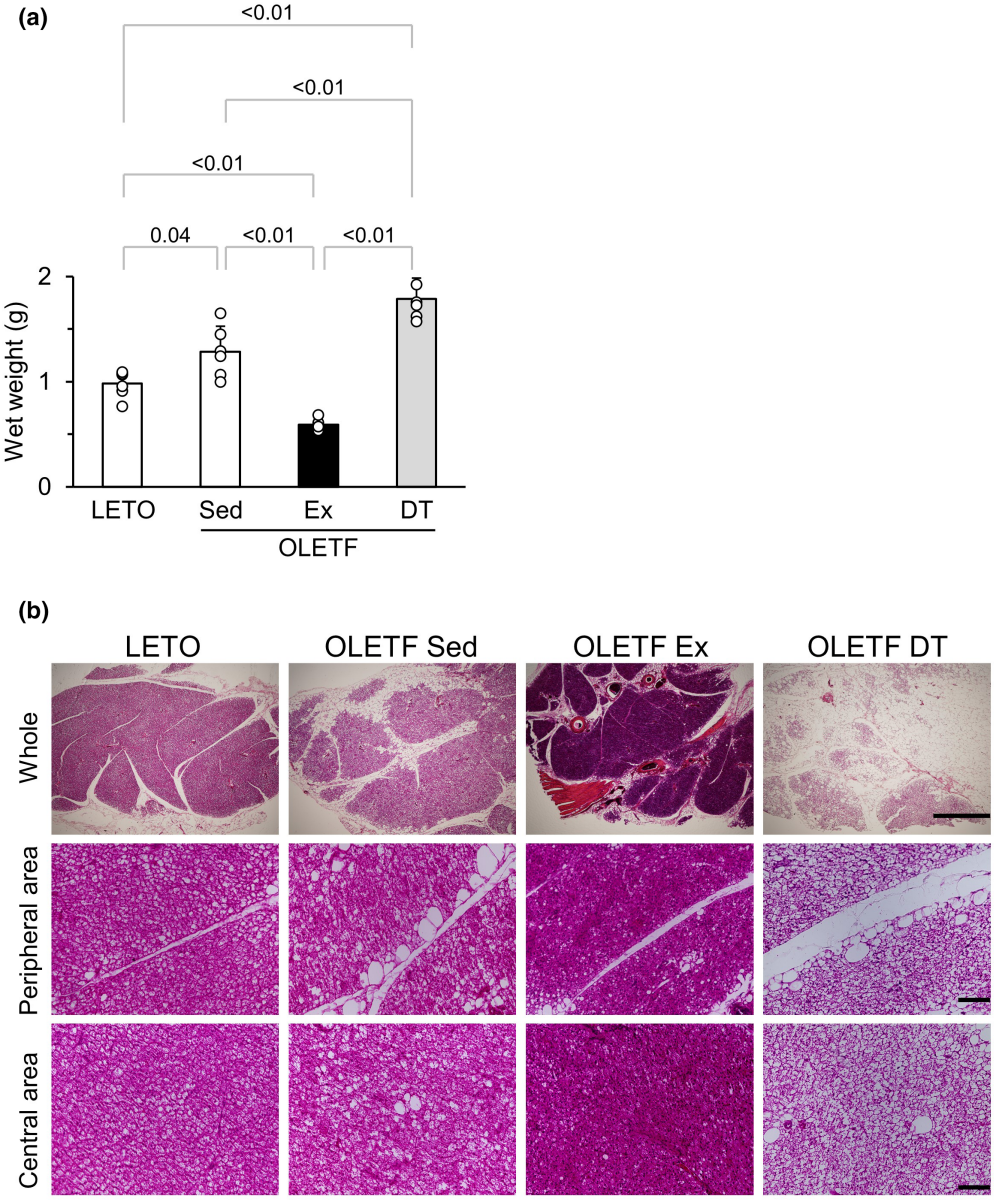
Influences of exercise and detraining at a young age on BAT. (a) Wet weight of BAT. (b) Representative sections of BAT stained with hematoxylin and eosin in the whole (upper), peripheral area (middle), and central area (lower) of the adipose lobule. Data are shown at 12 weeks of age. BAT, brown adipose tissue. Values represent means ± standard deviation. *p*‐Values were shown in the graph. Bars = 1000 μm (upper) and 100 μm (middle and lower).

Brown adipose tissue wet weight was significantly higher in the OLETF Sed group than in LETO rats (Figure 4a). The wet weight of BAT in the OLETF Ex group was significantly lower than that in the OLETF Sed group. Conversely, the wet weight of BAT in the OLETF DT group was significantly higher than that in the OLETF Sed group.

Whitened unilocular adipocytes were observed throughout the adipose lobule in the OLETF Sed and OLETF DT groups and rarely in LETO rats and the OLETF Ex group (Figure 4b). In the OLETF Sed group, although the size of whitened unilocular adipocytes was almost the same in the central area of the lobule, enlarged unilocular adipocytes were often observed in the peripheral area. However, in the OLETF DT group, enlarged unilocular adipocytes were observed not only in the peripheral area but also in the central area.

### 3.6 Liver

**FIGURE 5:**
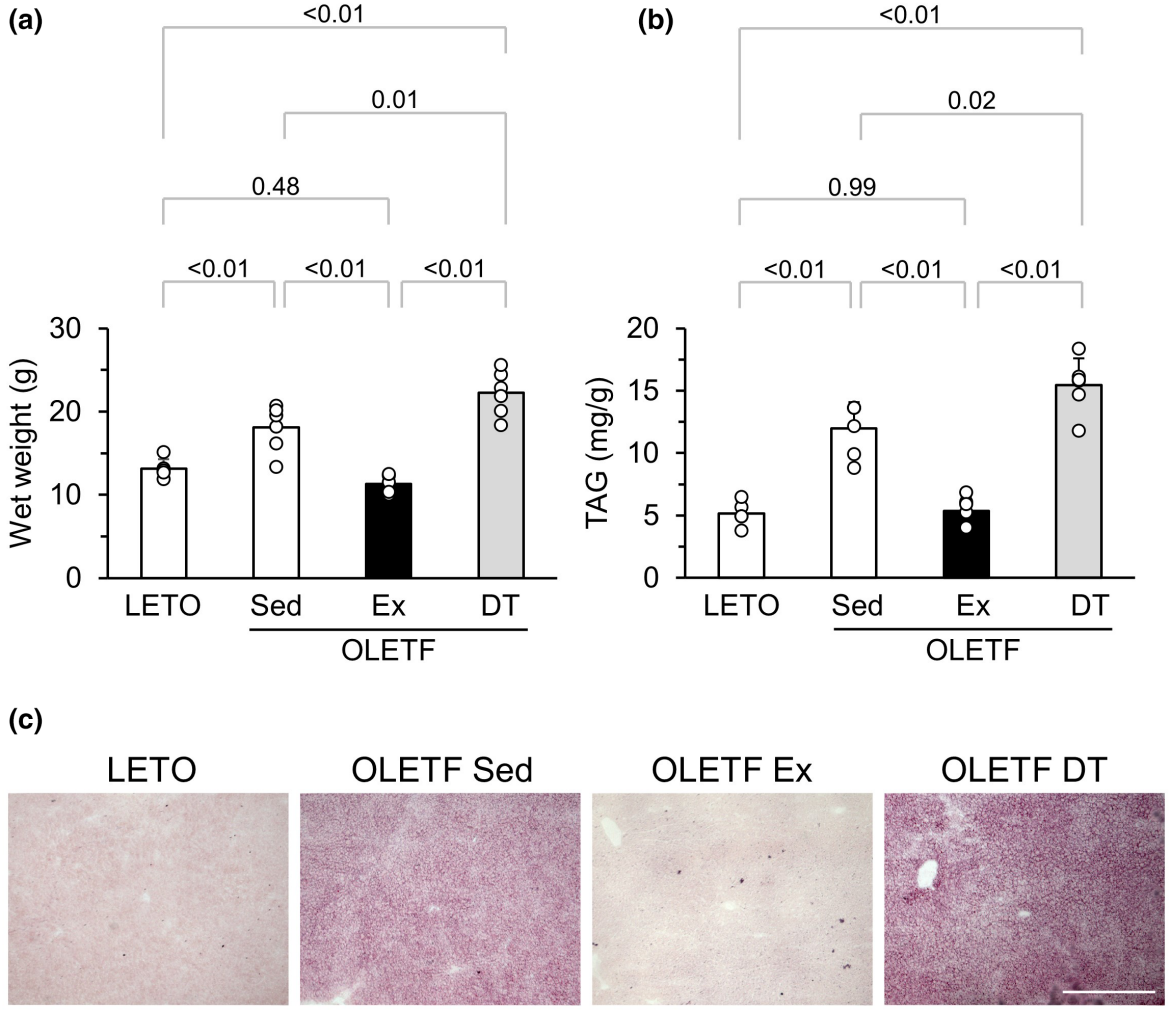
Influences of exercise and detraining at a young age on the liver. (a) Wet weight of the liver. (b) TAG levels in the liver. (c) Representative sections of the liver stained with Oil red O. Data are shown at 12 weeks of age. TAG, Triacylglycerol. Values represent means ± standard deviation. *p*‐Values were shown in the graph. Bar = 500 μm.

Liver wet weight was significantly higher in the OLETF Sed group than in LETO rats (Figure 5a). The liver wet weight in the OLETF Ex group was significantly lower than that in the OLETF Sed group. Conversely, liver wet weight was significantly higher in the OLETF DT group than in the OLETF Sed group.

Liver TAG levels were significantly higher in the OLETF Sed group than in LETO rats (Figure 5b). The liver TAG levels in the OLETF Ex group were significantly lower than those in the OLETF Sed group. Conversely, liver TAG levels in the OLETF DT group were significantly higher than those in the OLETF Sed group. Oil‐red O‐stained portions were observed throughout the hepatic lobule in the OLETF Sed group (Figure 5c). These portions were weakly stained in the OLETF Ex group but strongly stained in the OLETF DT group.

### 3.7 Pancreas

Obvious ectopic fat accumulation was not observed in either the exocrine or the endocrine portions of the pancreas in any of the groups (data not shown).

### 3.8 Gastrocnemius muscle

Although the stained portions of oil red O were occasionally found under the sarcolemma, obvious stained portions indicating intramuscular fat were not observed in any of the groups. Regarding ATPase and SDH activities, the gastrocnemius muscles were composed of type I, IIA, and IIB fibers, and no obvious differences in muscle fiber type distribution and muscle fiber size were observed between groups (data not shown).

### 3.9 Influences of food restriction during the detraining period (experiment 2)

Experiment 2 was performed to verify the effects of increased food intake. Hyperphagia was observed in the OLETF DT group during the detraining period. The rats in the OLETF DTFR group were subjected to pair feeding, and their daily food intake was adjusted to match that of the OLETF Sed group.

**FIGURE 6:**
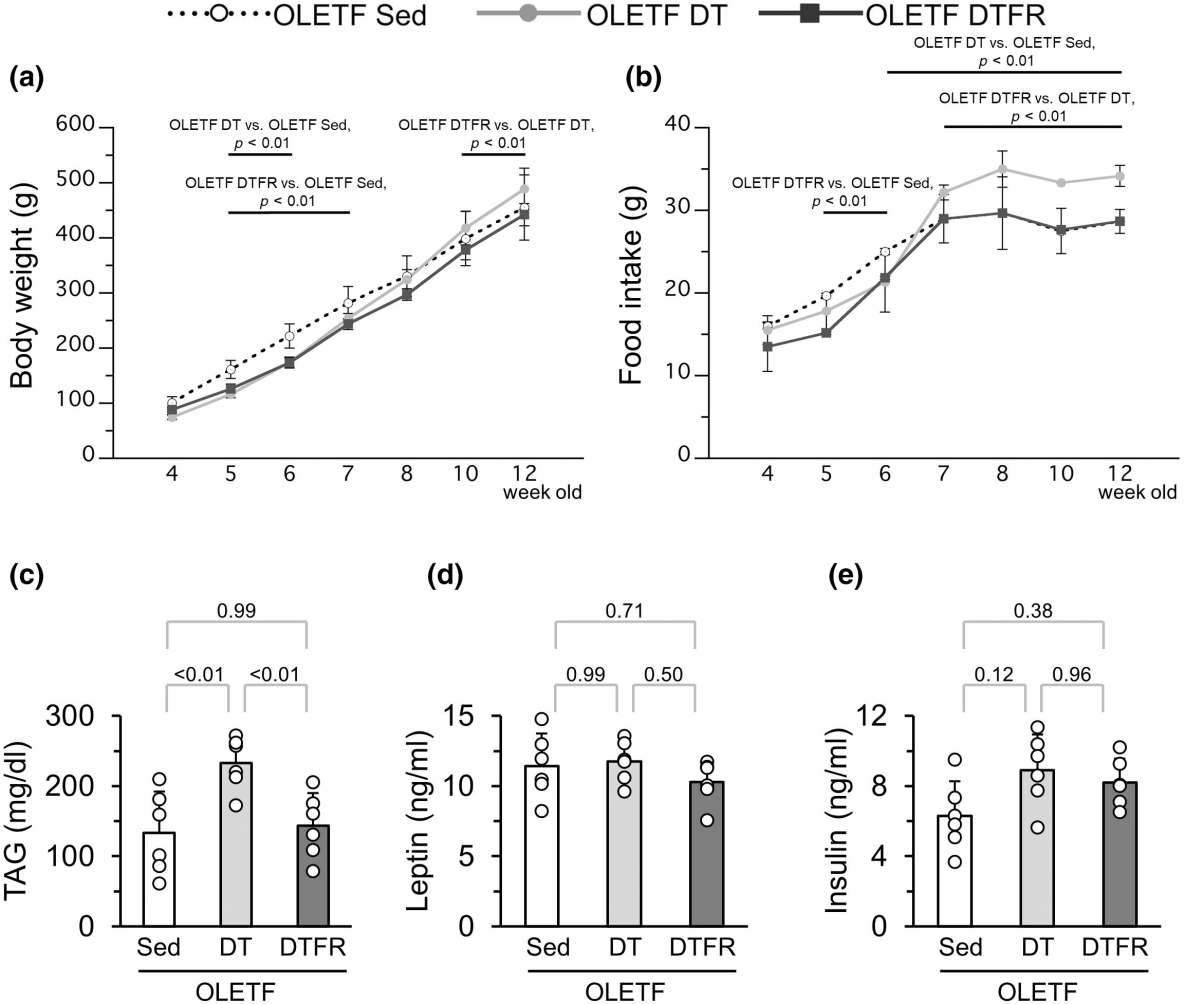
Influences of food restriction during the detraining period on body weight. (a) Changes in body weight. (b) Daily food intake in a rat. (c) Plasma TAG levels at 12 weeks of age. (d) Plasma leptin levels at 12 weeks of age. (e) Plasma insulin levels at 12 weeks of age. TAG, Triacylglycerol. Values represent means ± standard deviation. *p*‐Values were shown in the graph.

The body weights of the OLETF DT and OLETF DTFR groups were significantly lower than those of the OLETF Sed group between 5 and 6 weeks of age, that is, during the exercise period (Figure 6a). Although the body weight in the OLETF DT group gradually increased after 6 weeks of age (during the detraining period), the body weight in the OLETF DTFR group increased slowly during the detraining period. Food intake was significantly lower in the OLETF DT and OLETF DTFR groups than in the OLETF Sed group between 5 and 6 weeks of age, that is, during the exercise period. Food intake in the OLETF DTFR group was significantly lower than that in the OLETF DT group throughout the detraining period (Figure 6b).

Plasma TAG levels in the OLETF DT group were significantly higher than those in the OLETF Sed group (Figure 6c). The values in the OLETF DTFR group were significantly lower than those in the OLETF DT group. There was no significant difference between the values in the OLETF DTFR and OLETF Sed groups. The plasma leptin levels in the OLETF Ex group (1.5 ± 0.3 ng/mL) were significantly lower than those in the OLETF Sed group. However, there were no significant differences in plasma leptin levels between the OLETF Sed, OLETF DT, and OLETF DTFR groups (Figure 6d). In addition, the plasma insulin levels in the OLETF Ex group (2.4 ± 0.9 ng/mL) were significantly lower than those in the OLETF Sed group. However, there were no significant differences in plasma insulin levels between the OLETF Sed, OLETF DT, and OLETF DTFR groups (Figure 6e).

**FIGURE 7:**
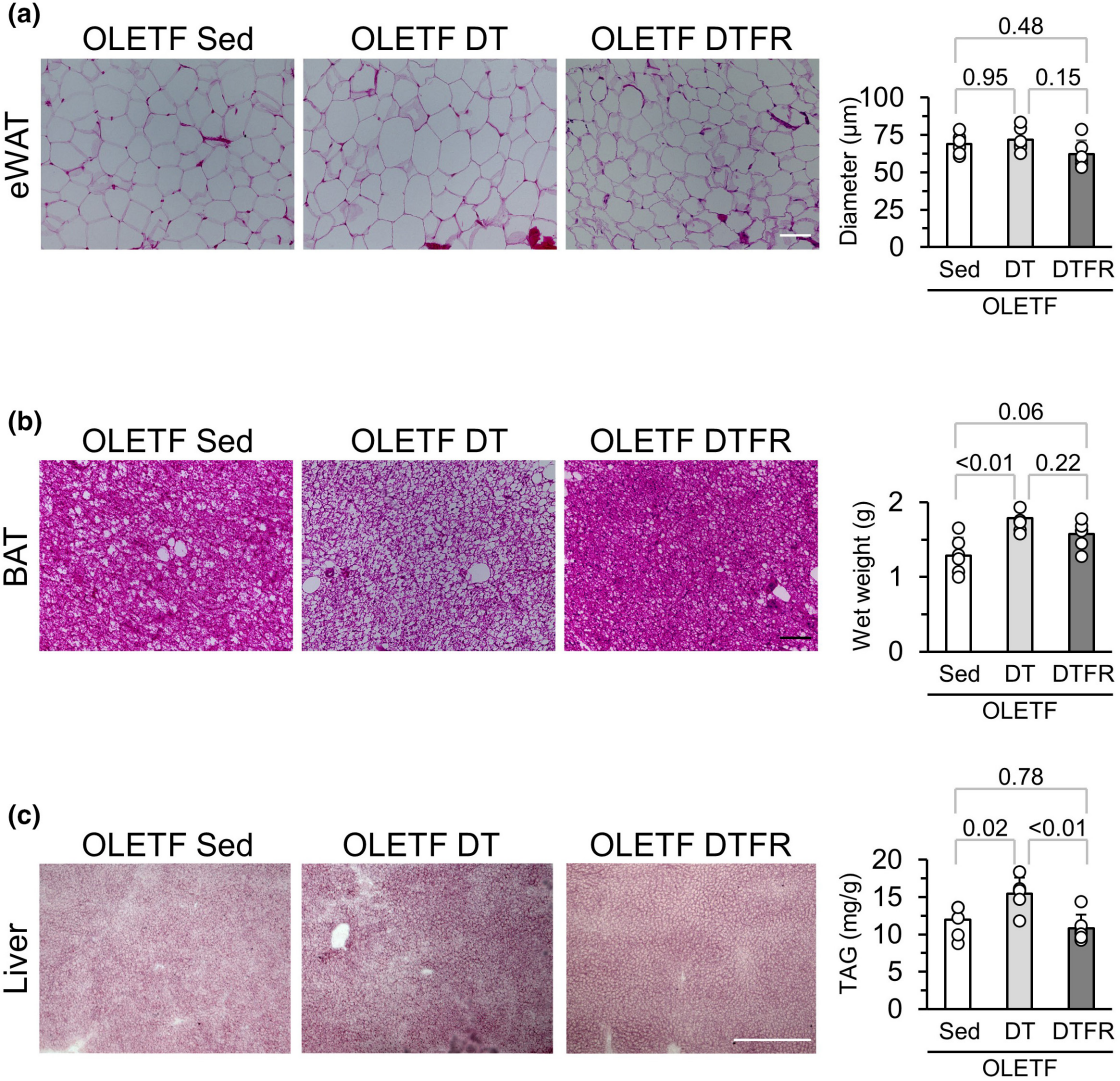
Influences of food restriction during the detraining period on fat accumulation in the organs. (a) Representative sections of eWAT stained with hematoxylin and eosin and mean adipocyte diameter. (b) Representative sections of BAT stained with hematoxylin and eosin in the central area of the adipose lobule and wet weight of BAT. (c) Representative sections of the liver stained with Oil red O and TAG levels in the liver. Data are shown at 12 weeks of age. eWAT, epididymal white adipose tissue; BAT, brown adipose tissue; TAG, Triacylglycerol. Values represent means ± standard deviation. *p*‐Values were shown in the graph. Bars = 100 μm (eWAT and BAT) and 500 μm (liver).

There were no significant differences in the mean diameter of adipocytes in eWAT between the OLETF Sed, OLETF DT, and OLETF DTFR groups (Figure 7a). Although BAT wet weight in the OLETF DT group was significantly higher than that in the OLETF Sed group, there were no significant differences in values between the OLETF DTFR and OLETF Sed groups (Figure 7b). Enlarged unilocular adipocytes located in the central area were observed in the OLETF DT group but not in the OLETF DTFR group. Although liver TAG levels in the OLETF DT group were significantly higher than those in the OLETF Sed group, the values in the OLETF DTFR group were significantly lower than those in the OLETF DT group (Figure 7c). There were no significant differences in the liver TAG levels between the OLETF DTFR and OLETF Sed groups. Oil‐red O staining was strong in the OLETF DT group but almost the same between the OLETF DTFR and OLETF Sed groups.

## 4 DISCUSSION

This study showed that detraining after early‐onset short‐term exercise accelerated triacylglycerolemia and extreme obesity at a young age. Hypertrophied adipocytes, indicating fat accumulation in eWAT, were observed equally and frequently in sedentary and detrained rats. Conversely, ectopic fat accumulation was predominantly observed in detraining‐induced extreme obesity and was characterized histologically by fatty liver and BAT whitening.

The cessation of regular exercise at a young age rapidly increases food intake during the detraining period. Additionally, hyperphagia caused by detraining after early‐onset short‐term exercise accelerates body weight gain during the detraining period. However, rapid hyperphagia and body weight gain during the detraining period have not always been reported in studies focusing on exercise and detraining. Linden et al. (2013) reported that no increase in food intake or body weight was found in their study. They reported results of detrained OLETF rats after voluntary wheel running for 12 weeks. Bi et al. (2005) revealed an increase in food intake and body weight during the detraining period, but the values did not reach the sedentary level in OLETF rats aged 20 weeks that performed exercise with a running wheel for 6 weeks. Therefore, the carry‐over effects of early‐onset exercise on appetite and body weight could depend on the exercise period (i.e., exercise between 4 and 16 weeks of age and detraining between 16 and 20 weeks of age in the study by Linden et al.; exercise between 4 and 6 weeks of age and detraining between 6 and 12 weeks of age in the present study) and implementation age (start from 8 weeks of age and detraining from 14 weeks of age in the study by Bi et al.; start from 4 weeks of age and detraining from 6 weeks of age in the present study). Interestingly, in a study by Schroeder et al. rapid increases in food intake and body weight were not observed during the detraining period of OLETF rats that performed short‐term voluntary wheel running (Schroeder et al., 2010). Although the exercise period was almost the same, the starting ages for the exercise were different in the study by Schroeder et al. and in the present study (i.e., exercise from immediately after weaning 3–6 weeks of age in the study by Schroeder et al. and exercise between 4 and 6 weeks of age in the present study). In the present study, none of the rats gained access to running wheels and were provided food and water ad libitum from 3 to 4 weeks of age after weaning. Therefore, the carry‐over effects of early‐onset exercise on appetite and subsequent body weight could depend not only on the duration of exercise but also on the implementation age. Patterson et al. (2008) reported that exercise for 3 weeks, but not 2 weeks, between 4 and 7 weeks of age prevented body weight gain during the detraining period. They also reported that food restriction immediately after weaning induced hypothalamic neurochemical dysfunction and a rapid increase in food intake after cessation of food restriction. Their results support the hypothesis that, in our study, the exercise duration was insufficient to control energy homeostasis during brain development. Food restriction with insufficient exercise duration could lead to a dysregulated appetite and induce hyperphagia and extreme obesity during the detraining period. We should consider the possibility of hyperphagia during the detraining period if we are expecting carry‐over effects of early‐onset short‐term exercise.

In the present study, excess fat accumulation in OLETF rats at 12 weeks of age was noticeable in eWAT, the liver, and BAT but not in the pancreas and skeletal muscle. When energy intake is greater than energy expenditure, the excess energy is stored primarily in white adipose tissues as TAG, resulting in obesity (Grundy, 2015). A rapid increase in fat accumulation was observed in the white adipose tissue of adult rats after cessation of regular exercise (Yasari et al., 2007). In the present study, hypertrophied adipocytes were frequently observed in eWAT during the detraining period, which was at the same level as that in the sedentary group. These results suggest that, even at a young age, white adipose tissue plays a role in accumulating excess fat owing to hyperphagia and inactivity during the detraining period. Interestingly, detraining after early‐onset short‐term exercise accelerated fat accumulation in the liver and BAT. Accumulation of fat in the liver is directly associated with increased food intake. Dietary carbohydrates directly reach the liver via the hepatic portal vein, and excess intake is converted into fatty acids and stored as intrahepatic TAG (Softic et al., 2016; Tappy & Lê, 2012). Additionally, although ectopic fat accumulation has been observed in the liver of some overfed obese rats regardless of dietary composition (Fujita et al., 2018; Hwang et al., 2018; Tatsumi et al., 2011), fatty pancreas and intramuscular fat have been observed in high‐fat diet‐induced obesity models (Shin et al., 2021; Zhou et al., 2014). In the present study, because all rats were fed a standard diet containing high carbohydrate content, hyperphagia during the detraining period could result in excess fat accumulation, particularly in the liver but not in the pancreas and skeletal muscle. Unlike fatty pancreas and intramuscular fat, BAT whitening induced by a high‐fat diet and a standard diet has been observed in obese models (Ku et al., 2016; Takaishi et al., 2021). BAT whitening is closely associated with capillary density. Since capillary density is higher in the periphery than in the central area of the adipose lobule of BAT (Shimizu et al., 2014), fat storage begins in the peripheral area, and once the limit is reached, it proceeds to continue in the central area. Lipid intake beyond the capacity induced by rapid hyperphagia during the detraining period in the OLETF DT group could result in excessive fat accumulation, not only in the peripheral area but also in the central area. In summary, increased food intake and physical inactivity during the detraining period after short‐term exercise at a young age cause rapid body weight gain and excess fat accumulation, particularly in the liver and BAT, depending on food composition and hemodynamics.

In tandem with the effectiveness of early‐onset exercise, the present study found that cessation of short‐term exercise at a young age caused subsequent hyperphagia and extreme obesity during the detraining period. However, food restriction during the detraining period prevented extreme obesity, with fatty liver and BAT whitening. Leptin and insulin serve as satiation and adiposity signals from the same neurons of the hypothalamic arcuate nucleus, which are secreted by the white adipose tissue and pancreas, respectively (Han et al., 2010). The plasma leptin levels of obese elementary school students showed lower values during the exercise period and higher values during the detraining period (Gutin et al., 1999). Additionally, in a study using OLETF rats at 20 weeks of age (Linden et al., 2013), lower plasma leptin levels were found in OLETF rats that exercised between 4 and 16 weeks of age and detrained at 16–20 weeks of age, which shows carry‐over effects of exercise on appetite and body weight during the detraining period. In the present study, lower plasma leptin levels were found in OLETF rats exercised between 4 and 12 weeks of age. However, there were no significant differences in plasma leptin and insulin levels between the OLETF Sed, OLETF DT, and OLETF DTFR groups, regardless of hyperphagia following extreme obesity during the detraining period. Accelerated appetite during the detraining period could result from changes in leptin and insulin resistance in OLETF rats.

The present study showed that detraining after early‐onset short‐term exercise caused hyperphagia and extreme obesity at a young age. Nevertheless, this study had some limitations. First, the experimental animals in the present study were only in male OLETF rats. This experimental design does not include female and animals without genetic predisposition to obesity such as LETO rats. This makes it difficult to clarify the influences of detraining on appetite, body weight, and adiposity. Second, all the rats were fed a standard diet. Generally, obesity is induced earlier and severer in high‐fat diet than in standard low‐fat diet. Variations in carbohydrate and fat content of the diet might affect the results after exercise period. Additionally, the experimental groups in the present study included both group‐housed and single‐housed rats. This makes it uncertain how much each rat ate in the group‐house. The differences in housing could contribute to the food intake during exercise and detraining periods. Further studies are required to compensate for insufficient investigation of physiological mechanism.

Although the mechanism of increased food intake during the detraining period has not yet been elucidated, food restriction during the detraining period prevents extreme obesity with fatty liver and BAT whitening. Regular physical exercise during childhood, without making the body weight loss, is recommended to prevent obesity‐related health problems in the future. However, in cases of exercise cessation, it is advisable to keep in mind the implementation age and the length of the exercise period while avoiding overeating during detraining.

## 5 CONCLUSION

Regular physical exercise prevented obesity at a young age. However, detraining after early‐onset exercise promoted hyperphagia, causing extreme obesity with fatty liver and histological whitening of brown adipose tissue. Although regular physical exercise at a young age is recommended to prevent obesity‐related health problems in the future, in cases of exercise cessation, it is advisable to keep in mind the implementation age and the length of the exercise period while avoiding overeating during detraining.

## FUNDING INFORMATION

This study was supported by a Grant‐in‐Aid for Scientific Research from the Japanese Ministry of Education, Culture, Sports, and Technology (19K11346 and 22K11393).

## CONFLICT OF INTEREST STATEMENT

There are no conflicts of interest to declare.

## ETHICS STATEMENT

This study was approved by the Institutional Animal Care and Use Committee of Hiroshima University (A19‐163) and conducted in accordance with the Hiroshima University Regulations for Animal Experimentation. All experiments were conducted in accordance with the National Institute of Health Guidelines for the Care and Use of Laboratory Animals.

## Data Availability

All data generated or analyzed during this study are included in this published article.

## References

[phy216055-bib-0001] Aggoun, Y. (2007). Obesity, metabolic syndrome, and cardiovascular disease. Pediatric Research, 61, 653–659. 10.1203/pdr.0b013e31805d8a8c 17426660

[phy216055-bib-0002] Ahmad, Q. I. , Ahmad, C. B. , & Ahmad, S. M. (2010). Childhood obesity. Indian Journal of Endocrinology and Metabolism, 14, 19–25. 21448410 PMC3063535

[phy216055-bib-0003] Bi, S. , Scott, K. A. , Hyun, J. , Ladenheim, E. E. , & Moran, T. H. (2005). Running wheel activity prevents hyperphagia and obesity in Otsuka long‐evans Tokushima fatty rats: Role of hypothalamic signaling. Endocrinology, 146, 1676–1685. 10.1210/en.2004-1441 15625240

[phy216055-bib-0004] Fujita, N. , Aono, S. , Karasaki, K. , Sera, F. , Kurose, T. , Fujino, H. , & Urakawa, S. (2018). Changes in lipid metabolism and capillary density of the skeletal muscle following low‐intensity exercise training in a rat model of obesity with hyperinsulinemia. PLoS One, 13, e0196895. 10.1371/journal.pone.0196895 29718998 PMC5931644

[phy216055-bib-0005] Grundy, S. M. (2015). Adipose tissue and metabolic syndrome: Too much, too little or neither. European Journal of Clinical Investigation, 45, 1209–1217. 10.1111/eci.12519 26291691 PMC5049481

[phy216055-bib-0006] Guthold, R. , Stevens, G. A. , Riley, L. M. , & Bull, F. C. (2020). Global trends in insufficient physical activity among adolescents: A pooled analysis of 298 population‐based surveys with 1·6 million participants. Lancet Child and Adolescent Health, 4, 23–35. 10.1016/S2352-4642(19)30323-2 31761562 PMC6919336

[phy216055-bib-0007] Gutin, B. , Ramsey, L. , Barbeau, P. , Cannady, W. , Ferguson, M. , Litaker, M. , & Owens, S. (1999). Plasma leptin concentrations in obese children: Changes during 4‐mo periods with and without physical training. The American Journal of Clinical Nutrition, 69, 388–394. 10.1093/ajcn/69.3.388 10075321

[phy216055-bib-0008] Han, J. C. , Lawlor, D. A. , & Kimm, S. Y. (2010). Childhood obesity. Lancet, 375, 1737–1748. 10.1016/S0140-6736(10)60171-7 20451244 PMC3073855

[phy216055-bib-0009] Hwang, Y. C. , Oh, D. H. , Choi, M. C. , Lee, S. Y. , Ahn, K. J. , Chung, H. Y. , Lim, S. J. , Chung, S. H. , & Jeong, I. K. (2018). Compound K attenuates glucose intolerance and hepatic steatosis through AMPK‐dependent pathways in type 2 diabetic OLETF rats. The Korean Journal of Internal Medicine, 33, 347–355. 10.3904/kjim.2015.208 28142230 PMC5840580

[phy216055-bib-0010] Jacobs, K. , Brouha, S. , Bettencourt, R. , Barrett‐Connor, E. , Sirlin, C. , & Loomba, R. (2016). Association of nonalcoholic fatty liver disease with visceral adiposity but not coronary artery calcification in the elderly. Clinical Gastroenterology and Hepatology, 14, 1337–1344. 10.1016/j.cgh.2016.01.010 26820400

[phy216055-bib-0011] Janssen, X. , Mann, K. D. , Basterfield, L. , Parkinson, K. N. , Pearce, M. S. , Reilly, J. K. , Adamson, A. J. , & Reilly, J. J. (2016). Development of sedentary behavior across childhood and adolescence: Longitudinal analysis of the Gateshead millennium study. International Journal of Behavioral Nutrition and Physical Activity, 13, 88. 10.1186/s12966-016-0413-7 27484336 PMC4971697

[phy216055-bib-0012] Kelley, G. A. , & Kelley, K. S. (2013). Effects of exercise in the treatment of overweight and obese children and adolescents: A systematic review of meta‐analyses. Journal of Obesity, 2013, 783103. 10.1155/2013/783103 24455215 PMC3886589

[phy216055-bib-0013] Ku, C. R. , Cho, Y. H. , Hong, Z. Y. , Lee, H. , Lee, S. J. , Hong, S. S. , & Lee, E. J. (2016). The effects of high fat diet and resveratrol on mitochondrial activity of brown adipocytes. Endocrinology and Metabolism, 31, 328–335. 10.3803/EnM.2016.31.2.328 27077216 PMC4923418

[phy216055-bib-0014] Lee, S. , Bacha, F. , Hannon, T. , Kuk, J. L. , Boesch, C. , & Arslanian, S. (2012). Effects of aerobic versus resistance exercise without caloric restriction on abdominal fat, intrahepatic lipid, and insulin sensitivity in obese adolescent boys: A randomized, controlled trial. Diabetes, 61, 2787–2795. 10.2337/db12-0214 22751691 PMC3478522

[phy216055-bib-0015] Lee, S. , Deldin, A. R. , White, D. , Kim, Y. , Libman, I. , Rivera‐Vega, M. , Kuk, J. L. , & Sandoval, S. (2013). Aerobic exercise but not resistance exercise reduces intrahepatic lipid content and visceral fat and improves insulin sensitivity in obese adolescent girls: A randomized controlled trial. American Journal of Physiology. Endocrinology and Metabolism, 305, E1222–E1229. 10.1152/ajpendo.00285.2013 24045865 PMC3840217

[phy216055-bib-0016] Linden, M. A. , Meers, G. M. , Ruebel, M. L. , Jenkins, N. T. , Booth, F. W. , Laughlin, M. H. , Ibdah, J. A. , Thyfault, J. P. , & Rector, R. S. (2013). Hepatic steatosis development with four weeks of physical inactivity in previously active, hyperphagic OLETF rats. American Journal of Physiology. Regulatory, Integrative and Comparative Physiology, 304, R763–R771. 10.1152/ajpregu.00537.2012 23467323 PMC3652080

[phy216055-bib-0017] Lloyd, L. J. , Langley‐Evans, S. C. , & McMullen, S. (2012). Childhood obesity and risk of the adult metabolic syndrome: A systematic review. International Journal of Obesity, 36, 1–11. 10.1038/ijo.2011.186 22041985 PMC3255098

[phy216055-bib-0018] Matsumoto, A. , Fujita, N. , Arakawa, T. , Fujino, H. , & Miki, A. (2014). Influence of electrical stimulation on calpain and ubiquitin‐proteasome systems in the denervated and unloaded rat tibialis anterior muscles. Acta Histochemica, 116, 936–942. 10.1016/j.acthis.2014.03.006 24745757

[phy216055-bib-0019] Mitsuhashi, T. , Yamada, C. , Iida, A. , Hiratsuka, N. , Inabe, F. , Araida, N. , Moriyama, K. , Sasamori, N. , Miyachi, M. , & Takahashi, E. (2011). Long‐term detraining increases the risk of metabolic syndrome in Japanese men. The Tokai Journal of Experimental and Clinical Medicine, 36, 95–99. 22167489

[phy216055-bib-0020] Morelli, M. , Gaggini, M. , Daniele, G. , Marraccini, P. , Sicari, R. , & Gastaldelli, A. (2013). Ectopic fat: The true culprit linking obesity and cardiovascular disease? Thrombosis and Haemostasis, 110, 651–660. 10.1160/TH13-04-0285 23884194

[phy216055-bib-0021] NCD‐RisC. Worldwide trends in body‐mass index, underweight, overweight, and obesity from . (1975). To 2016: A pooled analysis of 2416 population‐based measurement studies in 128·9 million children, adolescents, and adults. Lancet, 390(2627–2642), 2017–2642. 10.1016/S0140-6736(17)32129-3 PMC5735219 29029897

[phy216055-bib-0022] Nishimoto, Y. , & Tamori, Y. (2017). CIDE family‐mediated unique lipid droplet morphology in white adipose tissue and brown adipose tissue determines the adipocyte energy metabolism. Journal of Atherosclerosis and Thrombosis, 24, 989–998. 10.5551/jat.RV17011 28883211 PMC5656771

[phy216055-bib-0023] Olsen, R. H. , Krogh‐Madsen, R. , Thomsen, C. , Booth, F. W. , & Pedersen, B. K. (2008). Metabolic responses to reduced daily steps in healthy nonexercising men. JAMA, 299, 1261–1263. 10.1001/jama.299.11.1259 18349087

[phy216055-bib-0024] Padez, C. , Mourão, I. , Moreira, P. , & Rosado, V. (2005). Prevalence and risk factors for overweight and obesity in Portuguese children. Acta Paediatrica, 94, 1550–1557. 10.1080/08035250510042924 16303693

[phy216055-bib-0025] Paes, S. T. , Marins, J. C. , & Andreazzi, A. E. (2015). Metabolic effects of exercise on childhood obesity: A current view. Revista Paulista de Pediatria, 33, 122–129. 10.1016/j.rpped.2014.11.002 25662015 PMC4436964

[phy216055-bib-0026] Patterson, C. M. , Dunn‐Meynell, A. A. , & Levin, B. E. (2008). Three weeks of early‐onset exercise prolongs obesity resistance in DIO rats after exercise cessation. American Journal of Physiology. Regulatory, Integrative and Comparative Physiology, 294, R290–R301. 10.1152/ajpregu.00661.2007 17989137

[phy216055-bib-0027] Saponaro, C. , Gaggini, M. , Carli, F. , & Gastaldelli, A. (2015). The subtle balance between lipolysis and lipogenesis: A critical point in metabolic homeostasis. Nutrients, 7, 9453–9474. 10.3390/nu7115475 26580649 PMC4663603

[phy216055-bib-0028] Schroeder, M. , Shbiro, L. , Gelber, V. , & Weller, A. (2010). Post‐weaning voluntary exercise exerts long‐term moderation of adiposity in males but not in females in an animal model of early‐onset obesity. Hormones and Behavior, 57, 496–505. 10.1016/j.yhbeh.2010.02.008 20193686

[phy216055-bib-0029] Shimizu, I. , Aprahamian, T. , Kikuchi, R. , Shimizu, A. , Papanicolaou, K. N. , MacLauchlan, S. , Maruyama, S. , & Walsh, K. (2014). Vascular rarefaction mediates whitening of brown fat in obesity. The Journal of Clinical Investigation, 124, 2099–2112. 10.1172/JCI71643 24713652 PMC4001539

[phy216055-bib-0030] Shin, Y. , Lee, D. , Ahn, J. , Lee, M. , Shin, S. S. , & Yoon, M. (2021). The herbal extract ALS‐L1023 from Melissa officinalis reduces weight gain, elevated glucose levels and β‐cell loss in Otsuka long‐Evans Tokushima fatty rats. Journal of Ethnopharmacology, 264, 113360. 10.1016/j.jep.2020.113360 32918993

[phy216055-bib-0031] Softic, S. , Cohen, D. E. , & Kahn, C. R. (2016). Role of dietary fructose and hepatic de novo lipogenesis in fatty liver disease. Digestive Diseases and Sciences, 61, 1282–1293. 10.1007/s10620-016-4054-0 26856717 PMC4838515

[phy216055-bib-0032] Takaishi, K. , Oshima, T. , Eto, H. , Nishihira, M. , Nguyen, S. T. , Ochi, R. , Fujita, N. , & Urakawa, S. (2021). Impact of exercise and detraining during childhood on brown adipose tissue whitening in obesity. Metabolites, 11, 677. 10.3390/metabo11100677 34677392 PMC8540482

[phy216055-bib-0033] Tanaka, C. , Reilly, J. J. , & Huang, W. Y. (2014). Longitudinal changes in objectively measured sedentary behaviour and their relationship with adiposity in children and adolescents: Systematic review and evidence appraisal. Obesity Reviews, 15, 791–803. 10.1111/obr.12195 24899125

[phy216055-bib-0034] Tappy, L. , & Lê, K. A. (2012). Does fructose consumption contribute to non‐alcoholic fatty liver disease? Clinics and Research in Hepatology and Gastroenterology, 36, 554–560. 10.1016/j.clinre.2012.06.005 22795319

[phy216055-bib-0035] Tatsumi, K. , Sasaki, H. , Fujita, A. , Doi, A. , Kanaya, Y. , Furuta, H. , Nishi, M. , Tsuno, T. , Taniguchi, H. , & Nanjo, K. (2011). Effect of anti‐oxidants, Ricetrienol and α‐tocopherol, on adipocytokine abnormalities and fatty liver in Otsuka long‐Evans Tokushima fatty diabetic rats. Journal of Diabetes Investigation, 2, 186–192. 10.1111/j.2040-1124.2010.00090.x 24843482 PMC4014917

[phy216055-bib-0036] The, N. S. , Suchindran, C. , North, K. E. , Popkin, B. M. , & Gordon‐Larsen, P. (2010). Association of adolescent obesity with risk of severe obesity in adulthood. JAMA, 304, 2042–2047. 10.1001/jama.2010.1635 21063014 PMC3076068

[phy216055-bib-0037] Thompson, M. M. , Manning, H. C. , & Ellacott, K. L. (2013). Translocator protein 18 kDa (TSPO) is regulated in white and brown adipose tissue by obesity. PLoS One, 8, e79980. 10.1371/journal.pone.0079980 24260329 PMC3832377

[phy216055-bib-0038] van der Heijden, G. J. , Wang, Z. J. , Chu, Z. D. , Sauer, P. J. , Haymond, M. W. , Rodriguez, L. M. , & Sunehag, A. L. (2010). A 12‐week aerobic exercise program reduces hepatic fat accumulation and insulin resistance in obese, Hispanic Adolescents. Obesity, 18, 384–390. 10.1038/oby.2009.274 19696755

[phy216055-bib-0039] Yasari, S. , Dufresne, E. , Prud'homme, D. , & Lavoie, J. M. (2007). Effect of the detraining status on high‐fat diet induced fat accumulation in the adipose tissue and liver in female rats. Physiology & Behavior, 91, 281–289. 10.1016/j.physbeh.2007.03.012 17449070

[phy216055-bib-0040] Zhou, X. , Han, D. , Xu, R. , Li, S. , Wu, H. , Qu, C. , Wang, F. , Wang, X. , & Zhao, Y. (2014). A model of metabolic syndrome and related diseases with intestinal endotoxemia in rats fed a high fat and high sucrose diet. PLoS One, 9, e115148. 10.1371/journal.pone.0115148 25502558 PMC4263741

